# Diversity, distribution, and conservation status of *Macrobrachium* shrimp in freshwater ecosystems of Aceh, Indonesia

**DOI:** 10.14202/vetworld.2025.2377-2394

**Published:** 2025-08-21

**Authors:** Dedi Fazriansyah Putra, Muhammadar Abdullah Abbas, Tongku Nizwan Siregar, Daisy Wowor

**Affiliations:** 1 Graduate School of Mathematics and Applied Sciences, Universitas Syiah Kuala, Banda Aceh, 23111 Indonesia; 2 Department of Aquaculture, Faculty of Marine and Fisheries, Universitas Syiah Kuala, Banda Aceh, 23111 Indonesia; 3 Laboratory of Reproduction, Faculty of Veterinary Medicine, Universitas Syiah Kuala, Banda Aceh, 23111 Indonesia; 4 Museum Zoologicum Bogoriense, Research Center for Biosystematics and Evolution, National Research and Innovation Agency (BRIN), Cibinong, 16911 Indonesia

**Keywords:** Aceh, freshwater shrimp, habitat distribution, Indonesia, International union for conservation of nature, *Macrobrachium*, species diversity

## Abstract

**Background and Aim:**

Freshwater shrimps of the genus *Macrobrachium* play key ecological and socioeconomic roles in tropical freshwater ecosystems. However, their diversity, habitat associations, and conservation status remain understudied in Aceh Province, Indonesia. This study aimed to (1) assess the diversity and morphometric variation of *Macrobrachium* species, (2) map their distribution across lotic and lentic habitats in Aceh, (3) Assess the impact of environment variable on species composition and richness and (4) evaluate their conservation status based on the International Union for Conservation of Nature (IUCN) criteria and local environmental threats.

**Materials and Methods:**

Field sampling was conducted from September 2022 to December 2023 across 24 sites spanning 13 districts. Specimens were collected using hand nets and traps, and identified using morphological and morphometric criteria. Environmental parameters (temperature, pH, velocity, and substrate) were recorded, and biodiversity indices were calculated. Species distribution and conservation status were analyzed using local distribution (LD) indices and IUCN Red List categories.

**Results:**

A total of 1,303 *Macrobrachium* specimens representing 13 species were recorded. *Macrobrachium lanchesteri* was the most abundant and widely distributed species (LD = 50%), particularly in lentic habitats. Species such as *Macrobrachium pilimanus* and *Macrobrachium lar* showed narrow distributions. Lotic habitats supported higher species diversity (H’ = 1.28) compared to lentic ones. Environmental variables significantly influenced species presence. While 10 species were categorized as least concern, three species (*Macrobrachium australe, Macrobrachium esculentum, Macrobrachium mammillodactylus*) were unlisted by the IUCN, indicating data deficiency. Evidence of invasive species presence and habitat degradation was observed in several sites.

**Conclusion:**

Aceh hosts a diverse assemblage of *Macrobrachium* species, but they are vulnerable to anthropogenic disturbances and invasive species. The findings underscore the need for habitat conservation, sustainable fisheries management, and expanded monitoring–particularly for data-deficient species and those with narrow distributions. Molecular tools and long-term ecological monitoring are recommended for future research to better support regional conservation planning.

## INTRODUCTION

Indonesia’s freshwater ecosystems are home to a remarkable diversity of *Macrobrachium* shrimp species. However, their regional distribution, ecological functions, and conservation status remain poorly understood–particularly in Aceh Province. Globally, approximately 294 *Macrobrachium* species and subspecies have been documented [1]. The genus is of growing economic importance due to its large body size and high meat palatability, making it a valuable commodity in both capture fisheries and aquaculture systems [2–6].

Environmental parameters such as water temperature, pH, dissolved oxygen, and substrate composition are critical in shaping the distribution and abundance of *Macrobrachium* species [4, 7]. These shrimps typically inhabit rivers, lakes, and swamps–ecosystems that are highly sensitive to anthropogenic pressures. Human activities, including deforestation, pollution, and hydrological modifications, often lead to habitat degradation and reduced species distribution [8–10].

*Macrobrachium* species exhibit amphidromous life cycles, with some populations adapting to landlocked freshwater habitats [11–13]. This adaptive flexibility contributes to their broad geographic distribution and ecological diversity. However, infrastructure projects such as dam and irrigation system development can disrupt river connectivity and hinder shrimp migration and reproduction [14]. Moreover, contamination from domestic and industrial sources continues to degrade water quality, threatening the survival of local *Macrobrachium* populations [15].

According to the 2025 Indonesian International Union for Conservation of Nature (IUCN) Red List data, approximately 2.4% of aquatic species are classified as extinct or critically endangered, whereas 4.8% are endangered and 78.6% fall under the least concern (LC) category [16]. These figures underscore the urgent need for comprehensive ecological assessments and conservation planning.

Globally, research has highlighted the diversity and biogeographic distribution of *Macrobrachium* across various regions. India is noted for its high diversity and several endemic species confined to specific habitats [17–20]. Although *Macrobrachium* diversity is lower in Africa compared to Asia and South America, the continent still harbors distinct species [6, 21, 22]. Australia also supports a number of endemic *Macrobrachium* species [23, 24]. Southeast Asia, particularly, exhibits high genetic diversity within the genus, with species showing diverse larval development strategies–from full freshwater life cycles to reliance on brackish waters for larval rearing [25–28].

In Indonesia, extensive studies have revealed *Macrobrachium* diversity and phylogenetic relationships across various islands including Java, Borneo, Sulawesi, and Papua [29–39]. More recently, new species records have emerged from riverine and lacustrine habitats in Sumatra, further emphasizing the genus’s ecological richness in the region [40, 41].

Despite the ecological and economic importance of *Macrobrachium* species in tropical freshwater ecosystems, their diversity, distribution patterns, and conservation status in many regions of Indonesia remain poorly studied. While substantial taxonomic and ecological research has been conducted in regions such as Java, Borneo, Sulawesi, and Papua, Aceh Province, despite its ecological heterogeneity and extensive river systems, has received limited scientific attention. Previous records of *Macrobrachium* in Sumatra are fragmented, often based on anecdotal accounts or limited to specific habitats, without comprehensive ecological assessments or spatial distribution mapping. Furthermore, the environmental factors shaping the occurrence and abundance of *Macrobrachium* species across different habitat types (lotic vs. lentic) in Aceh remain unquantified. Conservation assessments have also been constrained by the absence of localized population data and habitat-specific threats, particularly regarding unlisted or data-deficient species within the IUCN framework. The increasing anthropogenic pressures-including habitat degradation, pollution, and the spread of invasive species-further underscore the need for systematic field surveys and ecological documentation to guide biodiversity management and conservation policies in Aceh’s freshwater systems.

This study aims to fill critical knowledge gaps by providing a comprehensive assessment of *Macrobrachium* diversity and distribution in the freshwater ecosystems of Aceh Province, Indonesia. Specifically, the objectives are to (1) identify and document *Macrobrachium* species using detailed morphological and morphometric analyses, (2) evaluate the distribution patterns across a range of lotic and lentic habitats using spatial and abundance metrics, (3) assess the influence of key environmental parameters, such as water temperature, pH, current velocity, and substrate type-on species composition and richness, and (4) determine the conservation status of identified species based on IUCN Red List criteria and local threat factors. Through integrative field sampling and ecological interpretation, this study seeks to provide baseline data essential for biodiversity monitoring, sustainable fisheries management, and the development of targeted conservation strategies for freshwater shrimp in Aceh.

## MATERIALS AND METHODS

### Ethical approval

All procedures involving animals adhered to the ethical standards of the Research Ethics Committee of Universitas Syiah Kuala (Approval No. 958/2015).

### Study period and location

**Figure 1:**
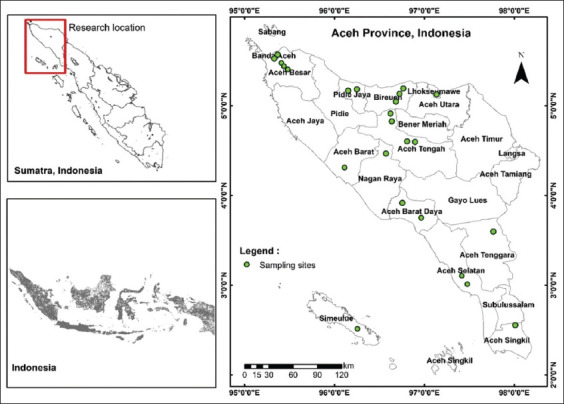
Map of the sampling sites in Aceh, Indonesia [Source: The map was generated using ArcGis 10.8., https://enterprise.arcgis.com/en/].

The study was conducted from September 2022 to December 2023. Freshwater shrimp sampling was conducted across 24 aquatic sites in Aceh Province, Indonesia. These sites spanned both lotic (flowing) and lentic (still) water systems across 13 administrative regions: Banda Aceh, Aceh Besar, Pidie Jaya, Bireuen, Lhokseumawe, Aceh Tengah, Aceh Barat, Aceh Barat Daya, Aceh Selatan, Nagan Raya, Aceh Singkil, Aceh Tenggara, and Simeulue (Figure 1).

### Sampling

**Figure 2:**
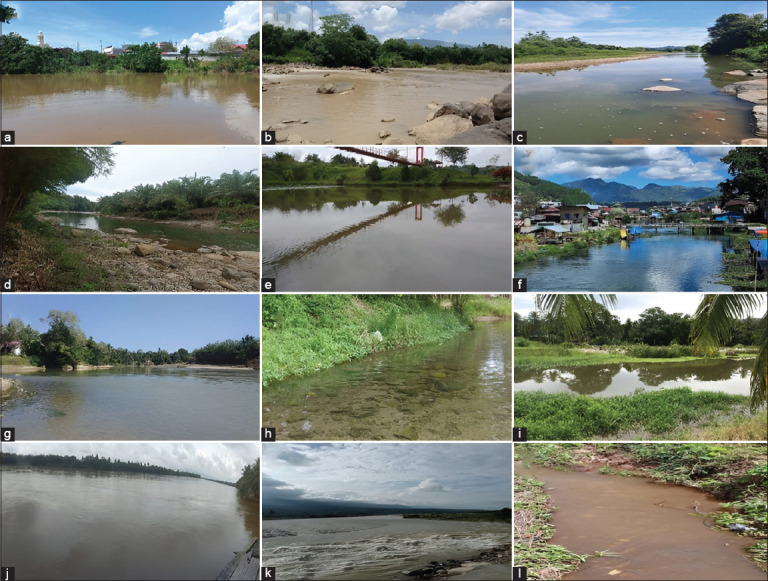
Representation of freshwater shrimp habitats in Aceh waters. (a) Banda Aceh, (b) Aceh Besar, (c) Pidie Jaya, (d) Bireuen, (e) Lhokseumawe, (f) Aceh Tengah, (g) Aceh Barat, (h) Aceh Barat Daya, (i) Aceh Selatan, (j) Aceh Singkil, (k) Aceh Tenggara, and (l) Simeulue.

A purposive sampling strategy was employed to ensure coverage of diverse habitat types–including pristine forested streams, anthropogenically influenced rivers, and isolated lentic water bodies (Figure 2). This approach prioritized environmental representativeness and logistical feasibility in remote areas with known or suspected *Macrobrachium* presence.

Sampling was conducted exclusively during the early and dry seasons to reduce hydrological variability and improve access to microhabitats. Stable water conditions during this period enhanced the accuracy of species abundance and distribution estimates. Site coordinates were recorded *in situ* using an Android-based Global Positioning System (GPS) application, based on the World Geodetic System 1984 (WGS84) reference datum, and detailed descriptions, including flow regime, substrate type, and vegetation cover, are presented in Table 1.

**Table 1:** Description of freshwater shrimp habitats in lentic and lotic areas of Aceh province.

No.	City/district	Sample location	Habitat description	Coordinates
1	Banda Aceh	Aceh River, Peunayong Village, Kuta Alam District	Muddy substrate, running water	95°19’05.3”E 5°33’37.0”N
		Leungpang River, Ateuk Pahlawan Village, Baiturrahman District	Muddy substrate, slow flowing water	95°19’48.2”E 5°33’12.4”N
2	Aceh Besar	Sabana River, Lamme Garot Village, Montasik District	Muddy and rocky substrate, flowing water	95°24’52.6”E 5°27’20.9”N
		Jeu River, Keude Village, Indrapuri District	Muddy and rocky substrate, flowing water	95°26’42.2”E 5°24’52.9”N
		Keuliling Lake, Keureuweng Village, Kuta Cut Gile District	Sandy substrate, mud, grass, lentic water	95°28’52.6”E 5°21’47.4”N
3	Pidie Jaya	Beuracan River, Beuracan Village, Meureudu District	Rocky substrate, gravel, sand, flowing water	96°13’03.6”E 5°13’01.8”N
		Trienggadeng Lake, Dee Peuduek Tunong Village, Trienggadeng District	Muddy substrate, lentic water	96°10’03.4”E 5°13’56.2”N
4	Bireuen	Krueng Teupin Mane	Rocky substrate, gravel, sand, flowing water	96°43’50.20”E 5° 8’0.45”N
		Paya Kareung Lake	Muddy substrate, lentic water	96°46’6.10”E 5°11’53.62”N
		Simpo River	Sandy rock substrate	96°42’45.94”E 5° 3’56.32”N
5	Lhokseumawe	Jeulikat Lake	Muddy substrate, lentic water	97° 6’18.46”E 5° 8’24.17”N
6	Aceh Tengah	Laut tawar lake	Sandy mud and rocky sand substrate accompanied by aquatic plants	96°55’24.49”E 4°36’44.59”N
		Totor bale river	Bush mud substrate	96°50’57.12”E 4°37’1.55”N
		Enang-enang river	Sandy rock substrate, shrubs	96°43’39.10”E 4°53’7.15”N
		Pante peusangan river	Sandy rock substrate	96°41’10.18”E 4°58’36.84”N
7	Aceh barat	Kawai XVI river	Mud substrate, sandy rock	96°10’52.24”E 4°14’32.98”N
8	Nagan raya	Beutong ateuh river	Sandy rock substrate	96°34’22.87”E 4°28’37.78”N
9	Aceh barat daya	Suak river	Sandy stone substrate, grass thicket	96°52’36.76”E 3°42’30.29”N
		Suak nihong river	Mud substrate	96° 8’21.70”E 4° 9’7.88”N
10	Aceh selatan	Geulumbuk river	Sandy rock substrate	97°20’34.69”E 3° 4’23.16”N
		Batee river	Mud substrate, grass bushes	97°19’18.37”E 3° 6’26.96”N
11	Aceh singkil	Lae Soraya River, Lentong Village, Kuta Baharu District	Sandy rock substrate	97°55’51.17”E 2°55’18.97”N
12	Aceh Tenggara	Alas River, Kayu Mentangur Village, Ketambe District	Sandy rock substrate	97°44’56.97”E 3°34’52.90”N
13	Simeulue	Irigasi River, Ganting Village, East Simeulue District	Sandy rock substrate	96°20’8.60”E 2°32’51.10”N

### Sampling design and ethical considerations

Wild *Macrobrachium* specimens were collected using scoop and trap nets. All specimens were preserved in 96% ethanol immediately after capture and transported to the Aquatic Health and Biotechnology Laboratory at Universitas Syiah Kuala for further processing. Sampling was limited to wild, non-protected freshwater environments, with no collection of endangered or protected species; hence, no collection permits were required under Indonesian biodiversity law.

### Measurement of environmental and habitat parameters

At each site, key physicochemical parameters were recorded to characterize the aquatic environment. Water temperature (°C) was measured using a digital thermometer (LUTRON DO–5510, Taiwan), and pH values were obtained using a portable pH meter (Hanna HI98107). Current velocity (m/s) was classified into five categories: very slow (<0.10), slow (0.10–0.25), moderate (0.25–0.50), fast (0.50–1.00), and very fast (>1.00), following standard stream hydrology guidelines.

Substrate types were categorized as sandy, muddy, rocky, or mixed, based on dominant surface features assessed within a 1 × 1 m quadrat. These classifications were supported by photographic documentation to ensure reproducibility and alignment with standard stream ecology protocols [42].

### Morphological identification and morphometric analysis

Shrimp species were identified under a stereo microscope (Olympus SZ61, Japan) using established taxonomic keys [12, 43–46]. Identification emphasized non-sexual morphological features to allow for consistent classification across all life stages and sexes. Juvenile and female specimens, which often lack distinct secondary traits, were cautiously identified using stable diagnostic characters such as the rostral formula, scaphocerite shape, and antennal carapace morphology.

Morphometric measurements, including total length (TL) and carapace length (CL), were taken using a digital caliper (Mitutoyo CD-6CS Japan, ± 0.01 mm accuracy). All measurements were performed on freshly euthanized specimens–immersed in cold water (4°C) for 2–5 min–to prevent tissue distortion before ethanol preservation. Identification was further supported by rostral characteristics and formulas [47]. Specimens were then deposited in the Museum Zoologicum Bogoriense and the Faculty of Marine and Fisheries laboratory for archiving.

### Biodiversity indices and statistical analysis

Species diversity and community structure were assessed using standard ecological indices. These included the Shannon–Wiener diversity index (H’), Pielou’s evenness (E), Simpson’s dominance index (C), and abundance (A). Abundance was quantified as the number of individuals per square meter (ind/m^2^), using data from standardized 1 × 1 m quadrats.

Diversity levels were categorized as follows: H’ < 1 (low), 1 ≤ H’ < 3 (moderate), and H’ ≥ 3 (high). Evenness (E) values <0.40 were interpreted as low, while values ≥0.75 indicated high uniformity in species distribution. A dominance index (C) approaching 1 reflected increasing prevalence of one or few species. The definitions, formulas, and units used are detailed in Table 2 [48–50]. Results were presented using descriptive and narrative interpretations supported by tables and figures.

**Table 2:** Biodiversity index formulas and variable definitions.

Index	Formula [48–50]	Description
Shannon-Wiener (H’)	H’ = −Σ (pi×ln pi)	*pi*: Proportion of individuals of species *i*; quantifies species diversity
Evenness (E)	E=H’/ln S	*S*: Number of species; measures uniformity of individual distribution
Dominance (C)	C = Σ (pi²)	Indicates dominance by one or a few species; higher *C*=higher dominance
Abundance (D)	D=N/A	*N*: Number of individuals; *A*: Area sampled (m²); expressed in ind/m²
Local distribution (LD)	LD = (n/N) × 100	*n*: Number of sites where species occurs; *N*: Total number of sampling sites; expressed in %

### Species distribution and conservation assessment

To evaluate the spatial distribution of species, the local distribution (LD) index was calculated as the proportion of sites where each species was recorded. High LD values (~100%) indicated widespread, generalist taxa, whereas low LD values suggested habitat specialists or declining populations. This index provided insights into ecological amplitude and habitat preferences.

Conservation status was assessed using the IUCN Red List database (www.iucnredlist.org). Species not currently listed or classified as data deficient were noted as requiring urgent further evaluation.

## RESULTS

### Species composition and abundance patterns

**Figure 3:**
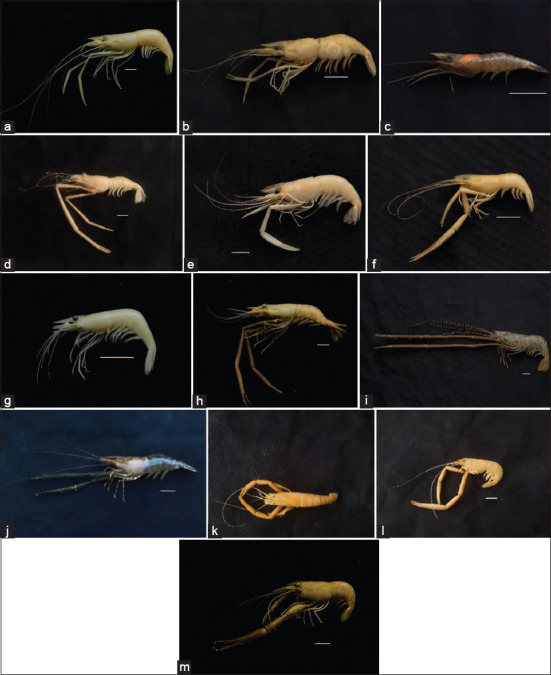
Freshwater shrimp species of the genus *Macrobrachium* occur in Aceh waters: (a) *Macrobrachium australe* Guérin-Méneville 1838, (b) *Macrobrachium Equidens* Dana 1852, (c) *Macrobrachium esculentum* Thallwitz 1891, (d) *Macrobrachium idae* Heller 1862, (e) *Macrobrachium latidactylus* Thallwitz 1891, (f) *Macrobrachium lanatum* Cai and Ng 2002, (g) *Macrobrachium lanchesteri* De Man 1991, (h) *Macrobrachium lar* Fabricius 1978, (i) *Macrobrachium mammillodactylus* Thallwitz 1892, (j) *Macrobrachium neglectum* De Man 1905, (k) *Macrobrachium pilimanus* De Man 1879, (l) *Macrobrachium placidum* De Man 1892, (m) *Macrobrachium scabriculum* Heller 1862. (scale: 1 cm).

A total of 1,303 *Macrobrachium* specimens were collected from 24 sampling sites across 13 regencies in Aceh Province. The surveyed habitats included 19 lotic (flowing) and 5 lentic (still) water bodies. Thirteen *Macrobrachium* species were identified: *Macrobrachium* *australe, Macrobrachium equidens, Macrobrachium esculentum, Macrobrachium idae*, *Macrobrachium latidactylus, Macrobrachium lanatum, Macrobrachium lanchesteri, Macrobrachium lar, Macrobrachium mammillodactylus, Macrobrachium neglectum, Macrobrachium pilimanus, Macrobrachium placidum*, and *Macrobrachium scabriculum* (Figure 3). Among these, *M. lanchesteri* was the most abundant species (987 individuals), followed by *M. esculentum* (101 individuals). The least abundant was *M. lar* and *M. scabriculum*, with only five individuals each (Table 3).

**Table 3:** Composition and distribution of freshwater shrimp species of the genus *Macrobrachium* in Aceh waters.

Species	Sampling sites																							

	A	B	C	D	E	F	G	H	I	J	K	L	M	N	O	P	Q	R	S	T	U	V	W	X
*Macrobrachium australe*	1	-	-	-	-	7	-	-	-	-	-	-	-	-	-	-	-	-	-	-	-	-	-	-
*Macrobrachium equidens*	-	4	-	-	-	-	-	3	-	-	-	-	-	-	-	-	-	-	-	-	-	-	14	2
*Macrobrachium escelentum*	-	4	-	-	-	-	-	-	-	-	-	-	-	-	1	96	-	-	-	-	-	-	-	-
*Macrobrachium idae*	-	-	-	-	-	-	-	-	-	-	-	-	-	-	-	-	-	-	-	-	1	14	-	-
*Macrobrachium latidactylus*	-	1	22	1	-	-	-	1	-	-	-	-	-	-	-	-	-	1	3	-	-	-	2	2
*Macrobrachium lanatum*	-	-	-	36	-	-	-	-	-	-	-	-	-	-	-	1	-	-	-	11	-	-	-	-
*Macrobrachium lanchesteri*	-	-	1	25	323	22	77	-	96	-	210	78	68	-	-	1	-	-	83	-	3	-	-	-
*Macrobrachium lar*	-	-	-	-	-	-	-	-	-	-	-	-	-	-	-	-	-	-	-	-	-	-	-	5
*Macrobrachium mammilodactylus*	4	-	-	-	-	-	-	-	-	-	-	-	-	-	-	-	-	1	-	-	-	-	-	7
*Macrobrachium neglectum*	-	4	3	-	-	-	-	-	-	-	-	-	-	-	-	-	-	3	-	3	-	-	-	-
*Macrobrachium pilimanus*	-	-	-	-	-	-	-	-	-	-	-	-	-	-	-	-	-	-	-	-	-	-	36	-
*Macrobrachium placidum*	-	-	-	-	-	-	-	-	-	-	-	-	-	-	1	-	-	2	-	-	-	-	14	-
*Macrobrachium scabriculum*	-	-	-	1	-	-	-	-	-	-	-	-	-	-	-	-	-	-	-	4	-	-	-	-

Letter code description (A) Aceh Peunayong River; (B) Leungpang River; (C) Sabana River; (D) Jeu River; (E) Keuliling Lake; (F) Beuracan River; (G) Trienggadeng Lake; (H) Krueng Teupin Mane; (I) Paya Kareung Lake; (J) Simpo River; (K) Jeulikat Lake; (L) Lake Laut Tawar; (M) Totor Bale River; (N) Enang-Enang River; (O) Pante Peusangan River; (P) Kawai River XVI; (Q) Beutong Ateuh River; (R) Suak River; (S) Suak Nihong River; (T) Geulumbuk River; (U) Batee River; (V) Lae Soraya River; (W) Alas River; and (X) Irigasi River. not available (−)

### Morphological identification and diagnostic features

Species identification was primarily based on morphological characteristics, particularly the number of postorbital teeth. Species such as *M. latidactylus, M. esculentum, M. placidum, M. pilimanus, M. scabriculum*, and *M. lanatum* had four or more postorbital teeth, whereas *M. lanchesteri*, *M. lar, M. neglectum, M. pilimanus*, and *M. australe* had three or fewer. Other diagnostic traits observed under a stereomicroscope included:

- Shape of the post-antennular carapace margin
- Rostral tooth base connection
- Margins of the anterior epistome
- Presence/absence of the preanal carina and the T4 median processes
- Proportions of the second pereiopod (carpus, merus, palm)
- Shape of the scaphocerite distal margin
- Size of the exopod spine
- Rostrum-to-CL ratio.

These combined traits supported accurate interspecific differentiation (Table 4).

**Table 4:** Morphological characteristics of the genus *Macrobrachium* in Aceh waters.

Species	Morphological characteristics
*Macrobrachium australe*	The rostrum is relatively long and extends beyond the tip of the *scaphocerite*, with evenly spaced carapace teeth. The post-antennular carapace margin is straight or nearly straight. The second pereiopod is elongate and asymmetrical in size; the carpus is shorter than the chela, but longer than the palm.
*Macrobrachium equidens*	The rostrum is long and curved upwards, equipped with nine dorsal and five ventral teeth. The chela of the second pereiopod has two toes covered by smooth, dense pubescence.
*Macrobrachium esculentum*	Rostrum is short and almost reaches the tip of the third segment of the *antennular peduncle*. The second pereiopod is asymmetrical and of varying size; the carpus is conical and shorter than the chela, palm, and merus. The entire segment is covered with small spines.
*Macrobrachium idae*	Rostrum has nine dorsal teeth. Second pereiopod with chela shorter than carpus. Surface of carapace, telson, and uropod tuberculated.
*Macrobrachium latidactylus*	The rostrum is short and sometimes extends beyond the tip of the third segment of the antennular stalk, but does not extend beyond the tip of the scaphocerite. The shape of the second pereiopod is asymmetrical and also varies in size. The major pereiopod has a wide chela with hairless fingers. While the minor pereiopod has a slender chela with many setae and long nails on the fingers that do not cover the surface. The telson and uropod do not have tubercles.
*Macrobrachium lanatum*	Rostrum has more than four postorbital teeth with a distinctly convex and rounded post-antennular carapace margin. Second pereiopod has a carpus longer than the palm and merus; the large second pereiopod has fingers longer than the palm. T8 segment in adult males shows anterior lobes that are close together posteriorly.
*Macrobrachium lanchesteri*	Rostrum convex proximally, with seven dorsal and three ventral teeth. The second pereiopod has fingers covered with smooth and dense pubescence.
*Macrobrachium lar*	Rostrum is short, reaching the distal end of the third segment of the antennular stalk, with a rostral formula of 1+7/2–6. The margin of the post-antennular carapace is rounded. The second pereiopod is slender and long, symmetrical in shape and size. All segments are covered with spines. The chela is longer than the carpus with an outer edge densely covered with spines. However, the carpus is shorter than the palm and is conical in shape.
*Macrobrachium mammilodactylus*	Rostrum is curved proximally and tapers distally. Rostrum spines point upwards with serrations spread along the rostrum and become denser proximally. There are about 8–14 dorsal serrations and 2–6 ventral serrations. The second cheliped on the right and left sides is symmetrical with a long size; the merus reaches the scaphocerite.
*Macrobrachium neglectum*	The rostrum has a straight dorsal margin, with eleven dorsal and two ventral teeth. The chela of the second major pereiopod are subcylindrical, with medium-sized spines that are erect and directed forward.
*Macrobrachium pilimanus*	The rostrum does not extend beyond the scaphocerite nor does it reach the tip of the third segment of the antennular peduncle. The carpus of the second pereiopod is shaped like a short claw. There is quite dense pubescence on the chela and second pereiopod.
*Macrobrachium placidum*	Rostrum has more than four postorbital teeth, with a distinctly convex and rounded post-antennular carapace margin. The bases of the rostral teeth are not connected to each other. The anterior edge of the epistome is rounded.
*Macrobrachium scabriculum*	Rostrum has more than four postorbital teeth, with a convex and rounded shape of the post-antennular carapace edge. The bases of the rostral teeth are not connected to each other. The second pereiopod has a carpus shorter than the length of the palm and merus. The large second pereiopod has fingers shorter or equal to the length of the palm. The T8 segment in adult males shows narrowly separated anterior lobes.

### Morphometric variation among species

Morphometric measurements included CL, TL, and total weight (TW). The largest individuals were recorded in *M. lar* (TL: 33.33–100.45 mm; TW: Up to 14.16 g) and *M. mammillodactylus* (TL: 37.85–99.64 mm; TW: Up to 9.50 g). In contrast, smaller-bodied species such as *M. lanchesteri*, *M. esculentum*, and *M. neglectum* exhibited limited size ranges and lower maximum body weights (e.g., *M. lanchesteri*: TL = 24.00–48.40 mm, BW = 0.11–0.81 g). *M. neglectum* showed the broadest TL range (9.12–99.65 mm) and the smallest recorded body weight (0.01 g), indicating a mixed age structure. Species such as *M. latidactylus*, *M. lanatum*, and *M. pilimanus* displayed intermediate growth profiles with average TL around 60 mm and BW of 3.5 g, suggestive of moderate ecological specialization (Table 5).

**Table 5:** Morphometric measurements of freshwater shrimps species of the genus *Macrobrachium*.

Species	Carapace length (mm)	Total length (mm)	Body weight (g)
*Macrobrachium australe*	9.87–35.90	25.14–83.59	0.13–6.75
*Macrobrachium equidens*	15.90–29.33	30.82–65.49	0.43–2.68
*Macrobrachium esculentum*	7.17–18.31	17.23–46.17	0.04–1.09
*Macrobrachium idae*	18.63–43.83	43.17–90.63	0.72–7.95
*Macrobrachium latidactylus*	13.23–27.68	27.76–57.96	0.19–3.30
*Macrobrachium lanatum*	5.36–32.89	16.46–60.44	0.06–3.46
*Macrobrachium lanchesteri*	4.4–12.5	24.00–48.4	0.11–0.81
*Macrobrachium lar*	12.33–45.93	33.33–100.45	0.39–14.16
*Macrobrachium mammilodactylus*	18.08–52.63	37.85–99.64	0.54–9.50
*Macrobrachium neglectum*	3.88–46.76	9.12–99.65	0.01–9.70
*Macrobrachium pilimanus*	10.21–34.77	35.05–54.31	0.46–1.57
*Macrobrachium placidum*	10.55–27.3	33.72–67.88	0.45–6.38
*Macrobrachium scabriculum*	10.61–30.99	37.90–47.83	0.91–2.26

### Habitat preferences and environmental conditions

Species demonstrated varying degrees of habitat specificity (Table 6):

**Table 6:** Habitat preferences, abundance, and conservation status of *Macrobrachium* species recorded in the study area.

Species	Habitat preferences	Abundance (ind/m^2^)	Conservation status
*Macrobrachium australe*	Lotic freshwater (rivers, streams; often in lower stretch habitats)	0.04	Unlisted
*Macrobrachium equidens*	Estuarine and brackish water	0.1	Least concern.
*Macrobrachium esculentum*	Lotic freshwater (often lowland with moderate flow)	0.99	Unlisted
*Macrobrachium idae*	Slow-flowing waters	0.075	Least concern.
*Macrobrachium latidactylus*	Freshwater rivers and streams (often in lowland zones)	0.16	Least concern.
*Macrobrachium lanatum*	Major river and streams with moderate to fast flow	0.245	Least concern.
*Macrobrachium lanchesteri*	Occur in lentic and lotic habitats (rivers or lakes)	4.94	Least concern.
*Macrobrachium lar*	Inhabites fast-flowing, oxygen-rich freshwater streams; larvae require brackish estuarine conditions.	0.025	Least concern.
*Macrobrachium mammilodactylus*	Freshwater streams (often associated with rocks or submerged vegetation)	0.06	Unlisted
*Macrobrachium neglectum*	Upland freshwater streams with fast current	0.065	Least concern.
*Macrobrachium pilimanus*	Fast current upland streams with rocky substrates	0.18	Least concern.
*Macrobrachium placidum*	Lowland rivers and streams	0.09	Least concern.
*Macrobrachium scabriculum*	Estuarine and brackish water habitats (tidal zones)	0.025	Least concern.

- *M. australe* and *M. lanatum* predominantly inhabit the lower reaches of major river systems.
- *M. pilimanus* were associated with fast-flowing upland streams
- *M. esculentum*, *M. latidactylus*, and *M. placidum* were primarily found in lowland rivers
- *M. scabriculum* and *M. equidens* occurred in brackish and estuarine environments
- *M. idae* favored organic-rich slow-flowing waters with muddy substrates
- *M. lar* inhabited fast-flowing, oxygen-rich freshwater streams, larvae require brackish estuarine conditions.
- *M. mammillodactylus* was typically found in vegetated, rocky stream beds.

Environmental parameters varied across sites:

- Temperature: 24.3°C (Lake Laut Tawar) to 33.5°C (Lake Keuliling)
- pH: 6.4 (Geulumbuk River) to 7.6 (Aceh Peunayong, Keuliling Lake)
- Current velocity: 0.048 m/s (Beuracan River) to 14.9 m/s (Alas River)
- Water depth: 0.2–1.5 m
- Alkalinity: 70–160 mg/L (Table 7).

**Table 7:** Environmental variables of lentic and lotic habitat in Aceh.

Environment variabels	Sampling sites																								Range

	A	B	C	D	E	F	G	H	I	J	K	L	M	N	O	P	Q	R	S	T	U	V	W	X	
Temperature (℃)	30.1	32.5	30	31	33.5	31.3	30	25.6	29.9	25.6	27.7	24.3	24.3	26.0	25.6	30	27	28.6	29.1	25.5	27.7	25.8	25.3	26.0	24.3–33.5
pH	7.6	7.2	7.2	7.2	7.6	7.2	6.8	7.5	7.2	7.5	7.2	7.2	7.2	6.8	6.8	6.2	6.2	6.5	7.2	6.2	6.4	6.9	7.4	6.8	6.2–7.6
Current (m/s)	0.09	0.09	0.2	9.7	0	0.13	0	0	-	-	-	0	14.2	0.06	8.68	0.10	0.10	0.11	0.09	0.09	0.05	9.2	14.9	0.09	0.05–14.9
Depth (m)	1	1	1	1.5	1.5	1	1.5	0.20	1	0.8	1.2	1.5	0.5	0.8	0.5	0.8	0.5	0.8	0.5	0.9	0.7	0.8	0.8	0.5	0.2–1.5
Alkalinity (ppm)	160	-	-	-	100	100	80	90	120		80	120	130	70	120	-	-	-	-	-	-	-	-	-	80–160

Letter code description (A) Aceh Peunayong River; (B) Leungpang River; (C) Sabana River; (D) Jeu River; (E) Keuliling Lake; (F) Beuracan River; (G) Trienggadeng Lake; (H) Krueng Teupin Mane; (I) Paya Kareung Lake; (J) Simpo River; (K) Jeulikat Lake; (L) Lake Laut Tawar; (M) Totor Bale River; (N) Enang-Enang River; (O) Pante Peusangan River; (P) Kawai River XVI; (Q) Beutong Ateuh River; (R) Suak River; (S) Suak Nihong River; (T) Geulumbuk River; (U) Batee River; (V) Lae Soraya River; (W) Alas River; and (X) Irigasi River.Irigasi

### Distribution patterns and LD indices (LDIs)

**Figure 4:**
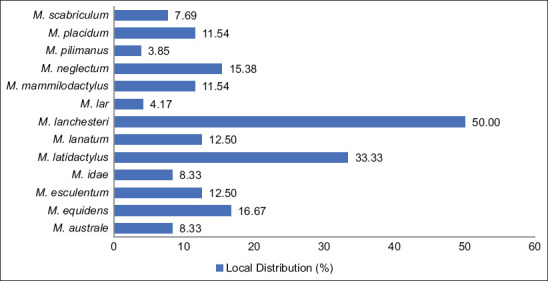
Percentages of *Macrobrachium* species recorded in the study area.

*M. lanchesteri* had the widest distribution (LD = 50.00%), found in both lentic and lotic waters across 12 sites. *M. latidactylus* was present in 8 lotic locations (LD = 33.33%). *M. equidens* and *M. neglectum* were each recorded at 4 sites (LD = 16.67%). *M. pilimanus* and *M. lar* exhibited the most restricted distributions, each found only in the Alas River and Irigasi River (LD = 4.17%) (Figure 4).

### Abundance and biodiversity index patterns

Lotic habitats supported higher *Macrobrachium* diversity than lentic ones.

**Figure 5:**
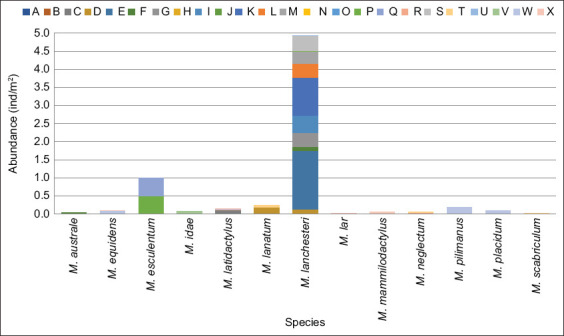
Graph showing the abundance index of freshwater shrimp species of the genus *Macrobrachium* in Aceh waters. The letter codes on the graph represent sampling locations as follows: (A) Aceh Peunayong River; (B) Leungpang River; (C) Sabana River; (D) Jeu River; (E) Keuliling Lake; (F) Beuracan River; (G) Trienggadeng Lake; (H) Krueng Teupin Mane; (I) Paya Kareung Lake; (J) Simpo River; (K) Jeulikat Lake; (L) Lake Laut Tawar; (M) Totor Bale River; (N) Enang-Enang River; (O) Pante Peusangan River; (P) Kawai River XVI; (Q) Beutong Ateuh River; (R) Suak River; (S) Suak Nihong River; (T) Geulumbuk River; (U) Batee River; (V) Lae Soraya River; (W) Alas River; and (X) Irigasi River.

- *M. lanchesteri* was most dominant in lentic habitats (4.94 ind/m^2^), followed by *M. esculentum* (0.99 ind/m^2^) and *M. lanatum* (0.25 ind/m^2^).
- *M. lar* and *M. scabriculum* had the lowest abundance (0.03 ind/m^2^) (Figure 5).

**Figure 6:**
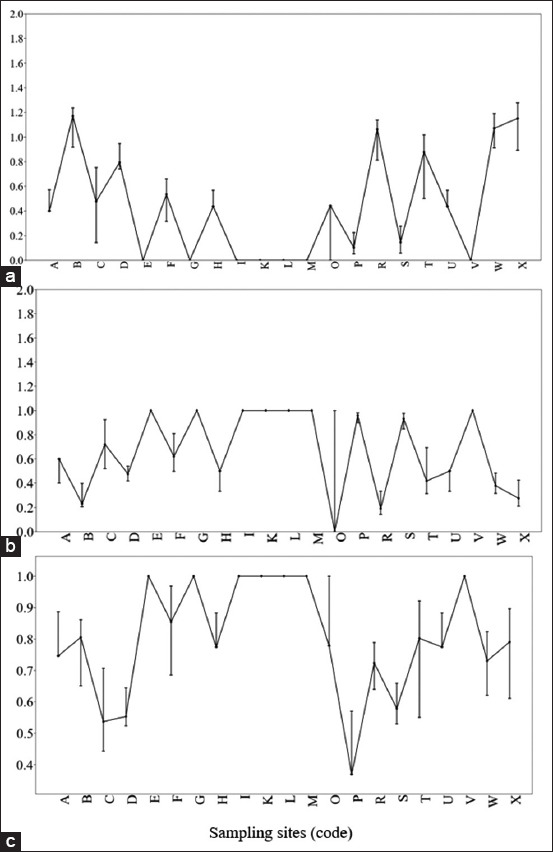
(a) Indices of diversity, (b) dominance, and (c) evenness of freshwater shrimp species of the genus *Macrobrachium* in Aceh waters. The letter codes on the graph represent the sampling locations as follows: (A) Aceh Peunayong River; (B) Leungpang River; (C) Sabana River; (D) Jeu River; (E) Keuliling Lake; (F) Beuracan River; (G) Trienggadeng Lake; (H) Krueng Teupin Mane; (I) Paya Kareung Lake; (J) Simpo River; (K) Jeulikat Lake; (L) Lake Laut Tawar; (M) Totor Bale River; (N) Enang-Enang River; (O) Pante Peusangan River; (P) Kawai River XVI; (Q) Beutong Ateuh River; (R) Suak River; (S) Suak Nihong River; (T) Geulumbuk River; (U) Batee River; (V) Lae Soraya River; (W) Alas River; and (X) Irigasi River.

The highest Shannon–Wiener diversity index (H’ = 1.28) was recorded in the Leungpang River and Suak River. Several lakes, including Keuliling and Laut Tawar, had a diversity index of 0.0. Across all sites:

- Mean H’: 0.50 (low diversity)
- Evenness (E): 0.82 (high)
- Dominance (C): 0.68 (moderate) (Figure 6).

### Conservation status of identified species

According to IUCN criteria:

- 10 species (*M. equidens*, *M. idae*, *M. latidactylus*, *M. lanatum*, *M. lanchesteri*, *M. lar*, *M. neglectum*, *M. pilimanus*, *M. placidum*, *M. scabriculum*) were listed as LC.
- 3 species (*M. australe*, *M. esculentum*, and *M. mammillodactylus*) were unlisted in the IUCN Red List, indicating potential data deficiency and the need for targeted conservation assessment (Table 8).

**Table 8:** Conservation status of freshwater shrimps species occur in Aceh province based on IUCN red list database.

Species	Common name	Native to Indonesia	IUCN status
*Macrobrachium australe*	-	Native	-
*Macrobrachium equidens*	Rough river shrimps	Native	Least concern
*Macrobrachium esculentum*	Sweet river shrimps	Native	-
*Macrobrachium idae*	Orana river shrimps	Native	Least concern
*Macrobrachium latidactylus*	-	Native	Least concern
*Macrobrachium lanatum*	-	Native	Least concern
*Macrobrachium lanchesteri*	-	Alien	Least concern
*Macrobrachium lar*	Monkey river shrimps	Native	Least concern
*Macrobrachium mammilodactylus*	-	Native	-
*Macrobrachium neglectum*	Java river shrimps	Native	Least concern
*Macrobrachium pilimanus*	Muff river shrimps	Native	Least concern
*Macrobrachium placidum*	-	Native	Least concern
*Macrobrachium scabriculum*	Goda river shrimps	Native	Least concern

- Not listed. IUCN=International union for conservation of nature

## DISCUSSION

### Biogeographic distribution patterns of *Macrobrachium*

*Macrobrachium* species exhibit wide-ranging biogeographic distributions across the Indo-Pacific. Some species, such as *M. lar* and *M. equidens*, have extensive geographic ranges–spanning from Australia to Tanzania and from India to the western Pacific, respectively (Table 9) [4, 7, 10, 16, 17, 28, 34, 36, 42, 44, 51–69]. These broad ranges are primarily facilitated by prolonged planktonic larval stages, which allow for long-distance dispersal via oceanic currents [4]. In contrast, species such as *M. pilimanus* and *M. lanchesteri* have more restricted distributions within Southeast Asia [7, 26, 51, 70], often due to abbreviated larval development and a fully freshwater lifecycle that limits their dispersal potential. In Southeast Asia, freshwater shrimps are predominantly represented by three families: Atyidae, Palaemonidae, and Alpheidae [71, 72]. In Indonesia, Palaemonidae and Atyidae dominate the freshwater shrimp fauna, with *Macrobrachium* being the most speciose and ecologically significant genus [4, 73, 74]. These shrimps inhabit a variety of ecosystems–including major rivers (e.g., Aceh, Peusangan, and Alas Rivers), lakes (e.g., Paya Kareung, Laut Tawar, Keuliling, and Jeulikat), and protected forest streams in Gunung Leuser National Park.

**Table 9:** Larval rearing habitats and distribution of 13 freshwater shrimps species in Aceh waters.

Species	Authority and year	Larvae nurturing habitat	Distribution in Indonesia	Distribution throughout the world	References
*Macrobrachium australe*	Guérin- Méneville, 1838	Sea water	Sulawesi, Bali, Jawa, Sumatera, Lesser Sunda Island	Fiji; French Polynesia (Marquesas, Tuamotu, Society island); India (Andaman island., Nicobar island); Madagascar; New Caledonia; Papua New Guinea (Papua New Guinea (main island group)); Réunion; Seychelles (Seychelles (main island group))	[4, 16, 36, 54]
*Macrobrachium equidens*	Dana, 1852	Brackish water	Jawa, Kalimantan, Bali, Sulawesi, Papua	Australia (Western Australia, Queensland, Northern Territory); Brunei Darussalam; Fiji; India (Orislandsa, Karnataka, Andhra Pradesh, Goa, West Bengal, Kerala, Maharashtra); Malaysia (Sarawak, Peninsular Malaysia); Mozambique; New Caledonia; Papua New Guinea (Papua New Guinea (main islandland group), North Solomons); Philippines; Singapore; Solomon islands (South Solomons); South Africa (KwaZulu-Natal); Taiwan, Province of China; Thailand; Vietnam	[4, 10, 16, 28, 55, 56]
*Macrobrachium esculentum*	Thallwitz, 1891	Brackish water	Sulawesi, Lesser Sunda island	Philippines; Taiwan, Province of China (Taiwan, Province of China (main island)	[4, 16, 54]
*Macrobrachium idae*	Heller, 1862	Brackish water	Sulawesi, Papua, Jawa	Australia (Queensland); Bangladesh; India (Tamil Nadu, Kerala, Karnataka); Kenya; Madagascar; Malaysia (Sabah); Papua New Guinea (Bismarck Archipelago); Seychelles (Seychelles (main island group)); Singapore; Thailand	[16, 28, 57–59]
*Macrobrachium latidactylus*	Thallwitz, 1891	Brackish water	Sulawesi, Papua, Lesser Sunda island	Australia (Queensland); China (Hainan); India (Andaman island., Nicobar island); Japan; Malaysia (Peninsular Malaysia); Philippines; Taiwan, Province of China (Taiwan, Province of China (main island); Thailand; Viet Nam	[4, 16, 55, 58]
*Macrobrachium lanatum*	Cai and Ng, 2002	Brackish water	Jawa, Sumatera	India; Indonesia (Jawa, Sumatera); Malaysia (Peninsular Malaysia); Myanmar (Myanmar (mainland)); Papua New Guinea (Papua New Guinea (main islandland group))	[4, 16, 17, 45]
*Macrobrachium lanchesteri*	De Man, 1911	Fresh water	Jawa, Kalimantan, Sulawesi, Sumatera	Brunei Darussalam; Lao People’s Democratic Republic; Malaysia (Peninsular Malaysia, Sabah); Singapore; Indonesia (Jawa, Kalimantan, Sulawesi, Sumatera), Thailand	[7, 16, 28, 45, 52, 60, 61]
*Macrobrachium lar*	Fabricius, 1798	Sea water	Sulawesi, Lesser Sunda island., Jawa, Papua	Australia (Queensland); Comoros; Fiji; French Polynesia (Marquesas); Guam; Japan (Kyushu); Kenya; Madagascar; Malaysia (Sarawak); Mauritius (Mauritius (main island), Rodrigues); Mozambique; New Caledonia; Northern Mariana islands; Papua New Guinea (Papua New Guinea (main island group)); Philippines; Réunion; Seychelles (Seychelles (main island group)); Taiwan, Province of China (Taiwan, Province of China (main island)); Tanzania	[16, 28, 53, 54,62–65]
*Macrobrachium mammilodactylus*	Thallwitz, 1892	Brackish water	Sulawesi, Sumatera	Mid China to Philippines, North Australia and New Guinea	[16, 66]
*Macrobrachium neglectum*	De Man, 1905	Brackish water	Sumatera	Malaysia (Peninsular Malaysia, Sarawak); Myanmar (Myanmar (mainland)); Singapore; Thailand	[16, 67]
*Macrobrachium pilimanus*	De Man, 1879	Fresh water	Sumatera, Jawa, Kalimantan	Brunei Darussalam; Malaysia (Peninsular Malaysia, Sabah); Singapore; Thailand	[4, 16, 43, 51]
*Macrobrachium placidum*	De Man, 1892	Brackish water	Sulawesi, Jawa, Sumatera, Papua	Western and central Southeast Asia, as well as the western Pacific margin	[4, 34, 68]
*Macrobrachium scabriculum*	Heller, 1862	Brackish water	Sumatera, Sulawesi	Bangladesh; India (Tripura, Tamil Nadu, Andhra Pradesh, Kerala, Orislandsa, Karnataka, West Bengal, Maharashtra); Kenya; Madagascar; Mozambique; Srilanka; Tanzania	[10, 17, 28, 45, 69]

The current study across 24 sites in Aceh revealed variable species richness. Three sites exhibited the highest diversity, each harboring four *Macrobrachium* species, while ten sites recorded only one species, indicating considerable spatial heterogeneity.

### Ecological plasticity and endemism

The observed distribution patterns align with broader biogeographic trends in *Macrobrachium*. Species such as *M. lar*, *M. equidens*, *M. australe*, *M. idae*, and *M. latidactylus* demonstrated wide geographic distributions, reflecting ecological adaptability and effective dispersal mechanisms. Conversely, *M. placidum*, *M. pilimanus*, and *M. lanchesteri* were regionally confined to Southeast Asia. Species such as *M. esculentum*, *M. neglectum*, and *M. mammillodactylus* had restricted national and global ranges. Notably, *M. mammillodactylus* exhibited a scattered but expansive distribution from mid-China to northern Australia. These findings underscore the genus spectrum of ecological specialization and highlight important implications for regional conservation and resource management.

### Emerging threat: Invasiveness of *M. lanchesteri*

*M. lanchesteri* has emerged as an invasive species in various regions due to its high ecological tolerance and competitive advantages. This study is the first to comprehensively report its widespread presence in Aceh’s freshwater habitats, particularly in lentic or slow-flowing systems such as swamps, rice fields, and irrigation canals [8, 12]. Key attributes contributing to its invasiveness include:

- Tolerance to environmental stress (e.g., low oxygen, pH fluctuations, and organic pollution)
- Rapid reproduction with short generation times
- Trophic plasticity enabling exploitation of diverse food sources.

These traits allow *M. lanchesteri* to dominate disturbed environments, outcompete native species, and disrupt ecological balances [52, 72, 75, 76]. Its spread poses significant threats to biodiversity through:

- Displacement of native shrimp
- Modification of sediment structure
- Increased turbidity and reduced aquatic vegetation
- Impaired nutrient cycling and detritus breakdown.

Documented ecological impacts include the decline of *M. sintangense* in West Java’s Lido River [52], and similar patterns have been observed globally, for example, *Cherax quadricarinatus* in Mexico [77] and *M. rosenbergii* in French Guiana [78]. In Aceh, the likely introduction pathway for *M. lanchesteri* involves inadvertent transfer through fish (e.g., tilapia) stocked into public waters.

### Habitat preferences and developmental strategies

Shrimp presence, abundance, and diversity are strongly influenced by habitat characteristics and reproductive strategies. Species in larger, connected water bodies benefit from higher habitat heterogeneity and access to brackish environments, promoting higher species richness [79–81].

*Macrobrachium* species follow either:

- Amphidromous development: Larvae require brackish/marine waters (e.g., *M. equidens, M. idae, M. latidactylus, M. lanatum, M. neglectum, M. placidum, M. australe, M. esculentum, M. mammilodactylus, M. scabriculum*, and *M. lar*)
- Abbreviated freshwater development: Larvae complete development in freshwater (e.g., *M. pilimanus*, *M. lanchesteri*).

Amphidromous species generally produce numerous small eggs with longer larval durations, while freshwater species produce fewer, larger eggs [4, 82–84]. For instance, *M. pilimanus* was found in the upstream reaches of the Alas River (Ketambe), far from brackish waters, highlighting its freshwater-adapted strategy. These differences in reproductive ecology affect dispersal capacity and influence habitat specificity.

### Environmental factors and anthropogenic pressures

Environmental variables such as temperature, pH, dissolved oxygen, and current velocity play a crucial role in maintaining freshwater shrimp assemblages [85, 86]. In Aceh, measured parameters remained within optimal ranges:

- Temperature: 24.3°C–33.5°C
- pH: 6.2–7.6
- Alkalinity: 80–160 ppm
- Flow velocity: 0.05–14.9 m/s.

Despite these favorable conditions, several rivers (e.g., Simpo, Beutong Ateuh, Enang-enang) showed an absence of shrimp, corroborated by local reports of population decline due to mining and destructive fishing (e.g., electrofishing, poisoning). These activities disrupt juvenile recruitment and population structure.

Combined with broader stressors, climate change, pollution, waste discharge, and dam construction, these anthropogenic impacts threaten the persistence of freshwater shrimp [4, 10, 87, 88]. The low overall diversity index observed across sites suggests a shift toward disturbance-tolerant or invasive species, consistent with patterns documented across Southeast Asia [88].

Encouragingly, community-led conservation frameworks in countries such as Thailand and Malaysia have demonstrated success [89, 90]. Similar initiatives, adapted to the Aceh context, could strengthen biodiversity conservation and freshwater ecosystem resilience.

### Conservation gaps and future research priorities

Globally, IUCN has evaluated 42 *Macrobrachium* species. Of the 13 species recorded in Aceh, 10 are classified as LC. However, *M. australe*, *M. esculentum*, and *M. mammillodactylus* are currently unlisted, reflecting significant data deficiencies. Among the 239 *Macrobrachium* species worldwide:

- 1 species is extinct
- 4 are critically endangered
- 4 endangered
- 7 vulnerable
- 1 near threatened
- 150 LC (least concern)
- 72 data deficient [16].

Although many species are considered low risk, these classifications often rely on outdated or incomplete data. There is a critical need for:

- Multi-seasonal, multi-habitat population monitoring
- Habitat quality and connectivity assessments
- Integration of molecular tools (e.g., DNA barcoding, eDNA)
- Evaluation of anthropogenic and climatic pressures.

Such research will inform conservation planning, enhance understanding of species distributions, and support long-term sustainability of freshwater shrimp in Aceh and beyond.

## CONCLUSION

This study provides the first comprehensive ecological and distributional assessment of *Macrobrachium* freshwater shrimp in Aceh Province, Indonesia. A total of 1,303 individuals representing 13 species were recorded across 24 sites encompassing both lotic and lentic habitats. The most abundant and widely distributed species was *M. lanchesteri*, particularly dominant in lentic systems, while species such as *M. pilimanus* and *M. lar* were rare and highly localized. Morphological and morphometric analyses revealed substantial interspecific variation in rostral dentition, carapace structure, and second pereiopod dimensions, which supported accurate species identification and provided insight into ecological strategies. Amphidromous and fully freshwater developmental modes were confirmed among the taxa, with distinct habitat preferences linked to flow regime, substrate type, and physicochemical parameters.

The results of this study offer essential baseline data for biodiversity conservation, aquaculture development, and freshwater ecosystem monitoring in Aceh. The identification of habitat specialists and disturbance-tolerant species enables more targeted habitat management and conservation prioritization. In addition, the detection of *M. lanchesteri* as a potentially invasive species underscores the need for early monitoring and intervention to mitigate its ecological impacts on native shrimp assemblages.

The strengths of the study lie in its broad geo-graphic coverage across 13 regencies and 24 ecologi- cally distinct sites, the integration of taxonomic, ecological, and morphometric data, and the documen-tation of previously unreported species in Aceh. However, the study has some limitations. Sampling was restricted to a single dry season, which may not reflect seasonal variability in shrimp communities. Molecular confirmation was not conducted for morphologically ambiguous individuals, and reproductive or behavioral traits were not assessed, which could provide deeper insights into population dynamics. Furthermore, rare species may have been underestimated due to accessibility issues and anthropogenic degradation at some sites.

Despite these limitations, the findings highlight that anthropogenic pressures–such as habitat degradation, pollution, invasive species, and unsustainable harvesting–are likely influencing the structure and richness of freshwater shrimp communities in the region. Future research should incorporate multi-seasonal surveys, apply molecular taxonomic tools, and explore larval ecology to better understand recruitment dynamics and dispersal pathways. Conservation strategies should focus on habitat restoration, regulation of destructive practices, and community-based monitoring to preserve native freshwater shrimp biodiversity. This study provides a critical foundation for future ecological and conservation planning in Aceh’s freshwater systems and contributes valuable knowledge to the regional understanding of *Macrobrachium* diversity and distribution.

## AUTHORS’ CONTRIBUTIONS

DFP and MAA: Designed the study, conducted field surveys, and collected shrimp samples. DFP and DW: Performed the laboratory examination. DFP, MAA, TNS, and DW: Data analysis, interpretation, and validation. All authors have read and approved the final manuscript.
